# Menstrual outcomes are frequently overlooked in von Willebrand disease trials

**DOI:** 10.1016/j.rpth.2026.106623

**Published:** 2026-05-05

**Authors:** Meaghan O’Donnell, Claire Kelly, Rezan Abdul Kadir, Roseline D’Oiron, Petra Elfvinge, Gaby Golan, Keith Gomez, Samantha C. Gouw, Laura Quintas, Karin P.M. van Galen, Michelle Lavin

**Affiliations:** 1 Irish Centre for Vascular Biology, School of Pharmacy & Biomedical Sciences, RCSI, Dublin, Ireland; 2 National Coagulation Centre, Department of Haematology, St. James’s Hospital, Dublin, Ireland; 3 Katharine Dormandy Haemophilia and Thrombosis Unit, Department of Obstetrics and Gynaecology, Royal Free Foundation Hospital and Institute for Women’s Health, University College London, London, UK; 4 Women & Girls with Bleeding Disorders + Working Group, European Association for Haemophilia and Allied Disorders, Brussels, Belgium; 5 Reference Centre for Hemophilia and Rare Bleeding Disorders, AP-HP, Bicêtre Hospital, University Paris-Saclay and UMR S1176 INSERM, Le Kremlin-Bicêtre, France; 6 Coagulation Unit, Department of Haematology, Karolinska University, Stockholm, Sweden; 7 Israel National Hemophilia Center, Sheba Medical Center, Israel; 8 Haemophilia Centre and Thrombosis Unit, Royal Free London NHS Foundation Trust, London, UK; 9 Amsterdam UMC location University of Amsterdam, Department of Pediatric Hematology, Amsterdam, the Netherlands; 10 European Haemophilia Consortium, Brussels, Belgium; 11 Van Creveldkliniek, University Medical Center Utrecht, Utrecht University, Utrecht, the Netherlands

**Keywords:** clinical trial, female, menstruation, treatment outcome, von Willebrand disease

## Abstract

**Background:**

Despite autosomal inheritance, females are disproportionately impacted by von Willebrand disease (VWD) due to heavy menstrual bleeding (HMB). HMB remains the most frequently reported and severe bleeding symptom in females with VWD. Nevertheless, interventional studies of VWD prophylaxis frequently fail to include or report menstrual-related outcomes.

**Objectives:**

This study evaluated the inclusion of menstrual and female-specific outcomes in clinical interventional trials of VWD prophylaxis.

**Methods:**

US (Clinicaltrials.gov), Canadian (https://www.canada.ca/en/health-canada/services/drugs-health-products/drug-products/health-canada-clinical-trials-database.html), and European (clinicaltrialsregister.eu) databases were searched for VWD interventional studies open to females with VWD aged >12 years between 2014 and 2024. Two reviewers assessed all studies, excluding those not focused on prophylaxis or duplicates. Inclusion criteria, outcomes, and publications were reviewed for menstrual-related data.

**Results:**

Initially, 42 interventional studies were identified; following exclusions, 10 studies remained. Only 2 of 10 (20%) studies incorporated menstrual inclusion criteria. Furthermore, 4 of 10 studies specifically excluded treated menstrual bleeding in their bleed eligibility criteria. Annualized bleeding rates were collected in 8 of 10 (80%) studies but menstrual outcomes in only 4 of 10 (40%). Most studies with results posted (*n* = 7) reported age (7/7) and sex (6/7) of participants; however, lack of overlapping data limited identification of females of menstrual age. Overall, identified females of menstrual age comprised 65 of 185 participants (35%) recruited.

**Conclusion:**

Clinical trials in VWD frequently use outcomes adapted from hemophilia studies (eg, annualized bleeding rates) rather than tailoring to the needs of females with VWD. Until we address this issue, we will lack clarity on optimal treatment, including the role of prophylaxis for the management of HMB in females with VWD.

## Essentials

• HMB is frequent experienced in women with VWD.
• Traditional clinical trial outcomes such as annualized bleeding rates fail to capture HMB.
• Treated menstrual bleeds were frequently excluded from eligibility criteria for VWD studies.
• Menstrual-specific outcomes were collected in only 40% of VWD interventional clinical trials.

## 1 Introduction

In the past 2 decades, there have been multiple major therapeutic advances for people with hemophilia, ranging from extended half-life products to novel rebalancing agents, factor [F]VIII mimetics, and gene therapies. In contrast, progress and options for people with von Willebrand disease (PwVWD) has been much slower, with only a single recombinant von Willebrand factor (VWF) product and no extended half-life products internationally. Central to all drug development are clinical trials, in which therapeutic efficacy is assessed against predefined outcomes. It is critical that both the population recruited and the outcomes selected are representative of those affected with the condition in order to translate trial outcomes into real-world clinical settings.

Recommendations regarding the use of prophylaxis are relatively recent for PwVWD [1], suggesting the use of long-term prophylaxis in PwVWD with a history of severe and frequent bleeds. There remains, however, a gap in guidance on the utilization of prophylaxis in the setting of menstrual bleeding (periodic prophylaxis).

For clinical trials of prophylaxis recruiting people with bleeding disorders, primary outcomes are rooted in reducing bleeding rate. However, bleeding patterns vary considerably between disorders, and enumerating bleeding events provides no appreciation of their duration or severity. In von Willebrand disease (VWD), females are disproportionately impacted due to impact of heavy menstrual bleeding (HMB) and account for the majority of PwVWD diagnosed internationally [2]. As HMB is the most frequently reported and severe bleeding symptom in females with VWD [3,4], it would be anticipated that menstrual outcomes are central to all clinical trials recruiting PwVWD. Subclassification of bleeds in studies, however, are often limited to the nature—traumatic or spontaneous—or the site of bleed. These assessments are largely meaningless in the context of menstruation, which is an anticipated bleed (rather than spontaneous or traumatic) with an expected event rate of 12 bleeds per year. As a result, there is uncertainty regarding the role or potentially benefit of prophylaxis in the management of HMB in PwVWD.

As part of our work for the European Association for Haemophilia and Allied Disorders Women and Girls + with Bleeding Disorders Working Group, we sought to compile clinical trial data on menstrual-related outcomes from all recent interventional trials recruiting PwVWD. We sought to provide an evidence base, if possible, regarding the role of prophylaxis on menstrual outcomes from which guidance could be developed.

## 2 Methods

Three clinical trial databases were searched to identify interventional clinical trials involving PwVWD—clinicaltrials.gov [5], the European Union Drug Regulating Authorities Clinical Trials Database [6], and Health Canada’s Clinical Trials Database [7] in the process outlined in our previous work [8]. Interventional trials from 2014 to 2024 were identified using the search terms (“prophylaxis”) AND (“von willebrand disease” OR “Willebrand” OR “VWD”) AND (“menstruation” OR “menstrual”), with the search conducted between September 4, 2024, and January 14, 2025. We excluded studies not focused on prophylaxis or only open to those aged < 12 years. Studies were evaluated by 2 reviewers, with all duplicates and those which prematurely terminated excluded. For each included study, the eligibility criteria and outcomes assessed were analyzed, recording VWD and female-specific data and contraceptive requirements. Outcomes for each study were compared against those defined in the CoreVWD prophylaxis and Women, Girls and Persons with the potential to menstruate (WGPPM) outcomes sets [9]. For those studies with results posted, the number and sex of participants ultimately enrolled were documented. If results were not available on the associated regulatory website, PubMed (https://pubmed.ncbi.nlm.nih.gov) was searched for linked publications using the clinical trial number to obtain recruitment data. Given that participants in clinical trials are referred to as males or females, we have used these terms throughout for consistency.

## 3 Results and Discussion

**Figure 1:**
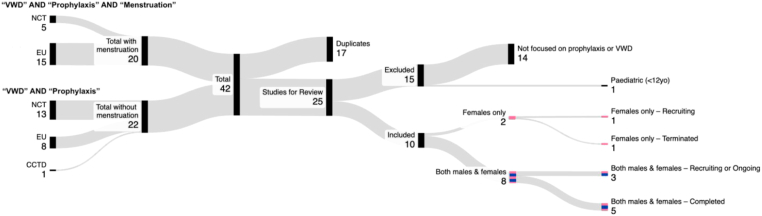
Sankey diagram outlining search terms and studies identified by site. CCTD, Canadian Clinical Trial Database [7]; EU, European Union Clinical Trial Database [6]; NCT, United States National Clinical Trial Database [5]; VWD, von Willebrand disease.

Using the search terms (“prophylaxis”) AND (“von willebrand disease” OR “Willebrand” OR “VWD”) AND (“menstruation” OR “menstrual”), 20 studies were initially identified. Recognizing that not all studies may include menstruation in their keywords, we also included studies identified using (“prophylaxis”) AND (“VWD” OR “Willebrand” OR “VWD”) alone, which yielded 22 studies (Figure 1) [5]. From these 42 identified interventional studies, 17 studies were duplicates and 15 were excluded (not focused on prophylaxis, *n* = 14; recruiting only those < 12 years, *n* = 1) (Figure 1). The remaining 10 studies that offered recruitment to females with VWD of menstrual age were reviewed. Two studies were focused specifically on menstrual bleeding and open to only females (“menstrual studies”) [10,11], with the remaining 8 studies (“general studies”) open to both males and females [[12], [13], [14], [15], [16], [17], [18], [19]].

### 3.1 Inclusion and exclusion criteria

Despite the frequency with which HMB impacts on females with VWD [3,20], menstrual criteria were frequently omitted from inclusion criteria. In fact, only the 2 studies focused on the treatment of HMB in females with VWD incorporated menstrual-related inclusion criteria (pictorial blood assessment chart [PBAC], >100; regular menses at least every 21 to 35 days; modified PBAC >100; and stable treatment for HMB for 3 cycles before entering the study and anticipated to remain unchanged) [10,11]. No other study included any form of menstrual eligibility criteria. Although 5 studies required treated bleeds in the last 6 to 12 months for inclusion in the trial [[12], [13], [14], [15],^18^], 4 studies specifically excluded treated menstrual bleeding in their bleed eligibility criteria [12,13,15,18].

We then explored the inclusion criteria regarding contraceptive requirements, if any, for all participants. The majority of studies (7/10) required females to engage in effective methods of contraception [10,[12], [13], [14], [15],^18^,^19^], whereas no studies had this requirement for males. In only 1 study was male partner sterilization included as a highly effective contraceptive option [19]. Use of hormonal therapy further limited eligibility, with 1 study explicitly excluding females who had used combined oral contraceptives or contraceptive implants within 3 months of the study start date [10], while another excluded those who were anticipated to start hormonal contraceptives or intrauterine device within the study period [11].

### 3.2 Outcome criteria

Although these studies recruited people only with VWD, the outcome criteria used were similar to that seen for hemophilia studies, with annualized bleeding rates in 8 of 10 studies, in all but the menstrual studies. Despite the diverse bleeding patterns of PwVWD, location of bleeding episodes (mucocutaneous vs joint bleeding) were included in the clinical trial record of only 4 of 10 studies [[13], [14], [15],^18^].

The CoreVWD outcomes set recently defined the outcomes that should be included in studies of PwVWD, divided into 3 domains—prophylaxis, WGPPM health, and perioperative treatment outcomes [9]. We retrospectively applied the WGPPM and prophylaxis outcomes set to our dataset to identify gaps in interventional studies, particularly for females with VWD.

**Figure 2:**
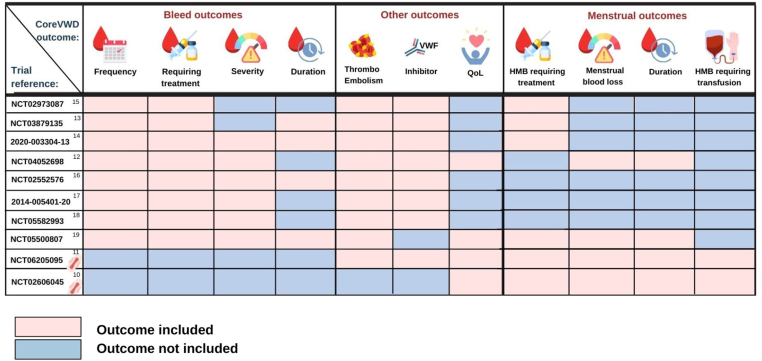
Outcomes for each included study analyzed according to CoreVWD outcome set: pink indicating that this outcome is recorded in this study; and blue, not included. Sanitary pad beside trial reference number indicates this is a menstrual-focused study. HMB, heavy menstrual bleeding; QoL, quality of life; VWD, von Willebrand disease; VWF, von Willebrand factor.

All general studies included bleed frequency and bleeds requiring treatment, with 7 of 8 studies reporting severity and 4 of 8 bleed duration (Figure 2). Thromboembolism and inhibitor formation were included as outcomes in the majority of general studies (Figure 2), but quality of life was infrequently collected (only 2/8 studies). In contrast, only the menstrual-focused studies consistently collected menstrual bleed outcome data, with HMB requiring treatment specifically listed as an outcome in only 4 of 8 general studies and menstrual blood loss or duration measured in only 2 of 8. HMB requiring transfusion was not included as an outcome in any general study.

At the time of analysis, enrollment data were available on only 7 of 10 studies overall (Table) [[10], [11], [12], [13], [14], [15], [16], [17], [18], [19],[21], [22], [23], [24], [25], [26]]. From these 7 studies, the sex of participants was not reported in 1 study, and in 3 others, the sex and age were reported separately, precluding assessment of menstrual age females. From review of clinical trial databases, in only 2 studies were females with VWD of menstrual age readily identified [10,15]. Review of associated publications identified further females potentially of menstrual age in 2 other studies [23,25], although determination of age was not possible in 1 study due to the method of reporting [25]. Overall, from the 185 PwVWD reported to have enrolled, we were able to identify only 65 females aged > 12 years (35%); even then, these data had to be extrapolated in many instances rather than clearly apparent. However, as the age and menopausal status of individuals are not reported, these may not necessarily reflect the number of females of menstrual age.

**Table:** Enrollment and participant data analyzed according to reported sex and age to identify females of menstrual age.

Clinical trial	Actual enrollment	Age of participants	Sex of participants	Females of menstrual age identified?	Associated publications
NCT02973087 [15]	23	≥ 18 y, *n* = 23	11 F, 12 M	Yes, 11 females	Primary [21]; secondary [22]
NCT03879135 [13] (continuation study [15])	38	Age available for *n* = 17 y: 12-17 y, *n* = 3; ≥ 18 y, *n* = 14	7 F, 10 M	At least 7 females, limited age/sex overlapping data	
2020-003304-13 [14]	24	< 12 y, *n* = 13; 12-17 y, *n* = 5	Not specified	No	
NCT04052698 [12]	43	Age available for *n* = 33: < 12 y, *n* = 9; 12-17 y, *n* = 6; **≥** 18 y *n* = 18	17 F, 26 M	7 females of menstrual age	Primary [23]; secondary: [24]
NCT02552576 [16]	14	< 12 y, *n* = 3; > 12 y, *n* = 11	8 F, 6 M	No, unable to ascertain age of females	Primary [25]
2014-005401-20 [17]	4	< 12 y, *n* = 1; **≥** 18 y, *n* = 3	2 F, 2 M	Unable to ascertain age of females, at least 1 female of menstrual age	
NCT05582993 [18]	Did not report				
NCT05500807 [19]	Did not report				
NCT06205095 [11]	Did not report				
NCT02606045 [10]	39	18-65 y, *n* = 39	39 F	Yes, 39	Primary [26]

Information correct as of October 24, 2025.

F, female; M, male.

Finally, looking at the associated publications [23,24] reporting results from the general studies, only 1 study reported on menstrual bleeding and the impact of treatment on menstrual outcomes for the females enrolled. In stark contrast, the publication on the first recombinant VWF replacement therapy includes reference to females only in the demographic table, despite females accounting for 48% of those enrolled [10]. Furthermore, reference to menstrual bleeding occurs only once, in the introduction as part of a description of the types of bleeding what PwVWD experience [21].

**Figure 3:**
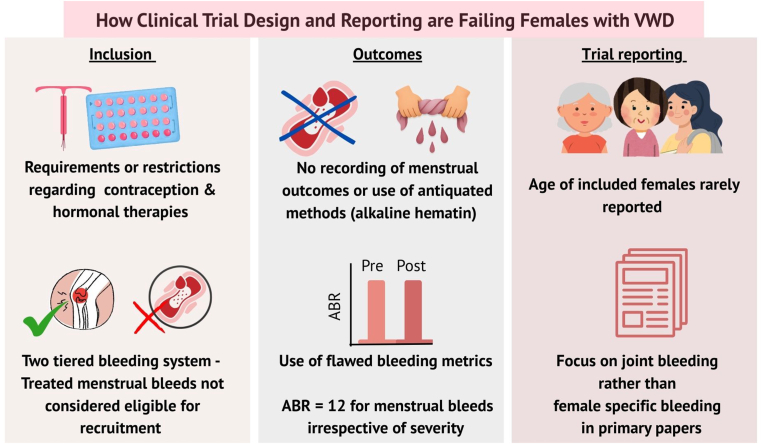
How clinical trial design is failing females with von Willebrand disease (VWD). ABR, annualized bleeding rate.

Cumulatively, these analyses of interventional clinical trials of prophylaxis recruiting PwVWD have highlighted a number of concerning findings (Figure 3). These studies were predominately based in high-income settings, where females account for 62% of those registered with VWD [2]. HMB is consistently the most frequently reported bleeding symptom in females with VWD [3,20,27]; despite this most studies failed to record or consider the impact of prophylaxis on menstruation. Clinical trial design, eligibility, and outcomes used largely mirrored studies for people with hemophilia, despite the different etiology of VWD and bleeding phenotype observed in PwVWD.

Inadvertently, the hemostasis community has allowed a 2-tiered bleed system to develop, where menstrual-related bleeding may be considered less important than other bleeds. This is emphasized by the requirement for treated bleeds in the eligibility criteria for trials, but with the exclusion of treated menstrual bleeds. The physiological or medical basis to demote treated menstrual bleeds as ineligible for recruitment is unclear. Furthermore, the exclusion of menstrual bleeding from trial publication or analysis is unacceptable from both the reporting pharmaceutical company and the publishing journal, particularly in the era of the Sex and Gender Equity in Research guidelines [28]. A bleeding episode in a person with a bleeding disorder should be taken seriously—excessive menstrual bleeding can no longer be overlooked and ignored.

The recommendations from recent evidence-based international guidelines for the management of VWD were significantly restricted due to the limited evidence base available in VWD. Transposition of hemophilia standards onto VWD trials risk creating knowledge gaps rather than meaningful data. Annualized bleed rate was originally developed to record predominantly joint and muscle bleeding in a hemophilia population, but annualized bleed rate provides no indication of bleed duration or severity. This has marked limitations in a VWD population, where the lack of weighting of severity may result in different bleeds (eg, 15-minute nosebleed, 14-day period, or massive gastrointestinal bleed), all resulting in the same recorded score (1 bleed). Regulatory constraints are often cited for the use of suboptimal methods in trial design; however, perpetuating their use limits understanding of the potential benefit of treatments for PwVWD.

While the CoreVWD proposed core outcome set is a step toward harmonization of VWD clinical trials, consensus on the optimal tools to measure and follow menstrual bleeding is lacking. Historically, hematin alkali methods were considered the gold standard; however, retention and extraction of menstrual blood from sanitary pads are challenging for participant acceptability [29]. PBACs are more convenient but require education on their use and may not be considered suitable by regulators [29]. Consensus recommendations from the hemostasis community are directly required to assist those involved in trial design and ensure standardization of approach.

Traditional clinical trial reporting segregates participants by age or sex, but overlapping data are often missing, making identification of females of reproductive age more challenging. As this is a subpopulation with specific bleeding issues, future trial reporting should endeavor to clearly delineate this subgroup. While concerns about reproductive toxicity are always more heightened for females of childbearing age, trial participation may also be impacted by the needs for or limitations on the use of contraceptive hormonal therapy. Additionally, the introduction of hormonal contraception for trial purposes may indirectly influence bleeding outcomes as hormonal therapies may alter menstrual bleeding profile, confusing efficacy analysis for the primary product being studied. In trials of novel agents, contraceptive options or use may be further restricted for females due to concerns regarding additive thrombotic risk. These issues merit careful consideration in trial planning and collaborative codesign with lived experience experts and clinicians essential.

Disappointingly, this review of clinical trials of prophylaxis for PwVWD from 2014 to 2024 identified little recorded or published data relating to menstrual bleeding in females with VWD. Future studies should aim to more clearly delineate their study population and ensure equity in both their eligibility criteria and outcomes for all types of bleeding and persons enrolled.

## References

[1] Connell N.T., Flood V.H., Brignardello-Petersen R., Abdul-Kadir R., Arapshian A., Couper S. (2021). ASH ISTH NHF WFH 2021 guidelines on the management of von Willebrand disease. Blood Adv.

[2] O’Sullivan J.M., Tootoonchian E., Ziemele B., Makris M., Federici A.B., Khayat Djambas C. (2023). Von Willebrand disease: gaining a global perspective. Haemophilia.

[3] Noone D., Skouw-Rasmussen N., Lavin M., van Galen K.P.M., Kadir R.A. (2019). Barriers and challenges faced by women with congenital bleeding disorders in Europe: results of a patient survey conducted by the European Haemophilia Consortium. Haemophilia.

[4] Lavin M., Aguila S., Schneppenheim S., Dalton N., Jones K.L., O’Sullivan J.M. (2017). Novel insights into the clinical phenotype and pathophysiology underlying low VWF levels. Blood.

[5] United States National Clinical Trial Database. http://www.clinicaltrials.gov.

[6] European Union Drug Regulating Authorities Clinical Trial Database. https://euclinicaltrials.eu/.

[7] Canadian Clinical Trial Database. https://www.canada.ca/en/health-canada/services/drugs-health-products/drug-products/health-canada-clinical-trials-database.html [Accessed September 4, 2024]

[8] O’Donnell M.J., Abdul Kadir R., van Galen K.P.M., Lavin M. (2025). Are women welcome in haemophilia trials?. Haemophilia.

[9] Clearfield E., Kim B., Ford S., Connell N.T., Santaella M.E., Lavin M. (2024). A core outcome set for prophylaxis and perioperative treatment of von Willebrand disease: the coreVWD initiative. Haemophilia.

[10] (2024). Minimize Menorrhagia in Women With Von Willebrand Disease (VWDMin) September.

[11] NCT06205095 A Pilot Crossover Trial of Prophylactic Wilate Compared to Placebo for Heavy Menstrual Bleeding in Patients with VWD (EMPOWER). NCT06205095.

[12] NCT04052698 Clinical Study to Investigate the Efficacy and Safety of Wilate During Prophylaxis in Previously Treated Patients With VWD. NCT04052698.

[13] NCT03879135 A Study of Recombinant Von Willebrand Factor (rVWF) in Pediatric and Adult Participants With Severe Von Willebrand Disease (VWD). NCT03879135.

[14] 2020-003304-13 A Phase 3, Prospective, Open-label, Uncontrolled, Multicenter Study on Efficacy and Safety of Prophylaxis with rVWF in Children Diagnosed With Severe von Willebrand disease. https://www.clinicaltrialsregister.eu/ctr-search/trial/2020-003304-13/NO.

[15] NCT02973087 rVWF in prophylaxis. NCT02973087.

[16] NCT02552576 Study of Voncento® in Subjects With Von Willebrand Disease. NCT02552576.

[17] 2014-005401-20 An Open-label, Multi-centre Study to Assess the Efficacy and Safety of Biostate® in Patients With von Willebrand’s Disease (VWD). https://www.clinicaltrialsregister.eu/ctr-search/search?query=2014-005401-20.

[18] NCT05582993 A Study of Vonicog Alfa (rVWF) in Children With Severe Von Willebrand Disease (vWD). NCT05582993.

[19] NCT05500807 Emicizumab for Severe Von Willebrand Disease (VWD) and VWD/Hemophilia A (BCDI-XII). NCT05500807.

[20] Lavin M., Aguila S., Dalton N., Nolan M., Byrne M., Ryan K. (2018). Significant gynecological bleeding in women with low von Willebrand factor levels. Blood Adv.

[21] Leebeek F.W.G., Peyvandi F., Escobar M., Tiede A., Castaman G. (2022). Recombinant von Willebrand factor prophylaxis in patients with severe von Willebrand disease: phase 3 study results. Blood.

[22] Leebeek F.W.G., Castaman G., Marier J.F., Özen G., Bhattacharya I., Zhang J. (2024). Exposure–response relationship between VWF/FVIII activity and spontaneous bleeding events following recombinant VWF prophylaxis in severe VWD. TH Open.

[23] Sidonio R.F., Boban A., Dubey L., Inati A., Kiss C., Boda Z. (2024). von Willebrand factor/factor VIII concentrate (Wilate) prophylaxis in children and adults with von Willebrand disease. Blood Adv.

[24] Kiss C., Boda Z., Khayat C.D., Boban A., Dubey L., Sidonio R.F. (2025). Efficacy of regular prophylaxis with a plasma-derived von Willebrand factor/factor VIII concentrate with a 1:1 activity ratio in reducing heavy menstrual bleeding in girls/women with von Willebrand disease. AJOG Glob Rep.

[25] Miesbach W., Halimeh S., Platokouki H., Podolak-Dawidziak M., Zdziarska J., Korczowski B. (2024). An open-label, multi-centre, post-marketing study to assess the efficacy and safety of a plasma-derived VWF/FVIII concentrate in patients with von Willebrand disease. Haemophilia.

[26] Ragni M.V., Rothenberger S.D., Feldman R., Nance D., Leavitt A.D., Malec L. (2023). Recombinant von Willebrand factor and tranexamic acid for heavy menstrual bleeding in patients with mild and moderate von Willebrand disease in the USA (VWDMin): a phase 3, open-label, randomised, crossover trial. Lancet Haematol.

[27] Olsson A., Elfvinge P., Zetterberg E., Myrin-Westesson L. (2025). Prevalence and impact of heavy menstrual bleeding in women with von willebrand disease across age groups: a retrospective study. Haemophilia.

[28] Heidari S., Babor T.F., De Castro P., Tort S., Curno M. (2016). Sex and gender equity in research: rationale for the SAGER guidelines and recommended use. Res Integr Peer Rev.

[29] Quinn S.D., Higham J. (2016). Outcome measures for heavy menstrual bleeding. Womens Health (London).

